# A terrestrial vertebrate palaeontological review of Aldabra Atoll, Aldabra Group, Seychelles

**DOI:** 10.1371/journal.pone.0192675

**Published:** 2018-03-28

**Authors:** Julian P. Hume, David Martill, Richard Hing

**Affiliations:** 1 Bird Group, Department of Life Sciences, Natural History Museum, Akeman St, Tring, Herts, United Kingdom; 2 School of Earth and Environmental Sciences, University of Portsmouth, Portsmouth, Hampshire, United Kingdom; Indiana University Bloomington, UNITED STATES

## Abstract

The Pleistocene vertebrate assemblage of Aldabra Atoll has been comparatively well studied. Three Upper Pleistocene fossil localities have been described yielding birds, reptiles and terrestrial molluscs. Those of Bassin Cabri and Bassin Lebine on Ile Picard are undated but must be in excess of 136,000 YBP, whereas Point Hodoul on Malabar Island is *circa* 100,000 YBP. Aldabra was seemingly completely submerged between deposition of the Ile Picard and Point Hodoul deposits, resulting in local faunal extinctions. Here we present the results of an ongoing study of fossil material collected on Ile Picard in 1987, which reveals a more diverse assemblage than previously realised. Notable discoveries are an *Ardeola* heron, three Procellariformes, tropic-bird *Phaethon*, gull *Larus*, rail *Dryolimnas*, harrier *Circus* and owl *Tyto*, plus evidence of recolonisation of the atoll by some seabirds, rail, harrier, owl, giant tortoises and lizards after the Ile Picard/Point Hodoul submergence event.

## Introduction

**Fig 1:**
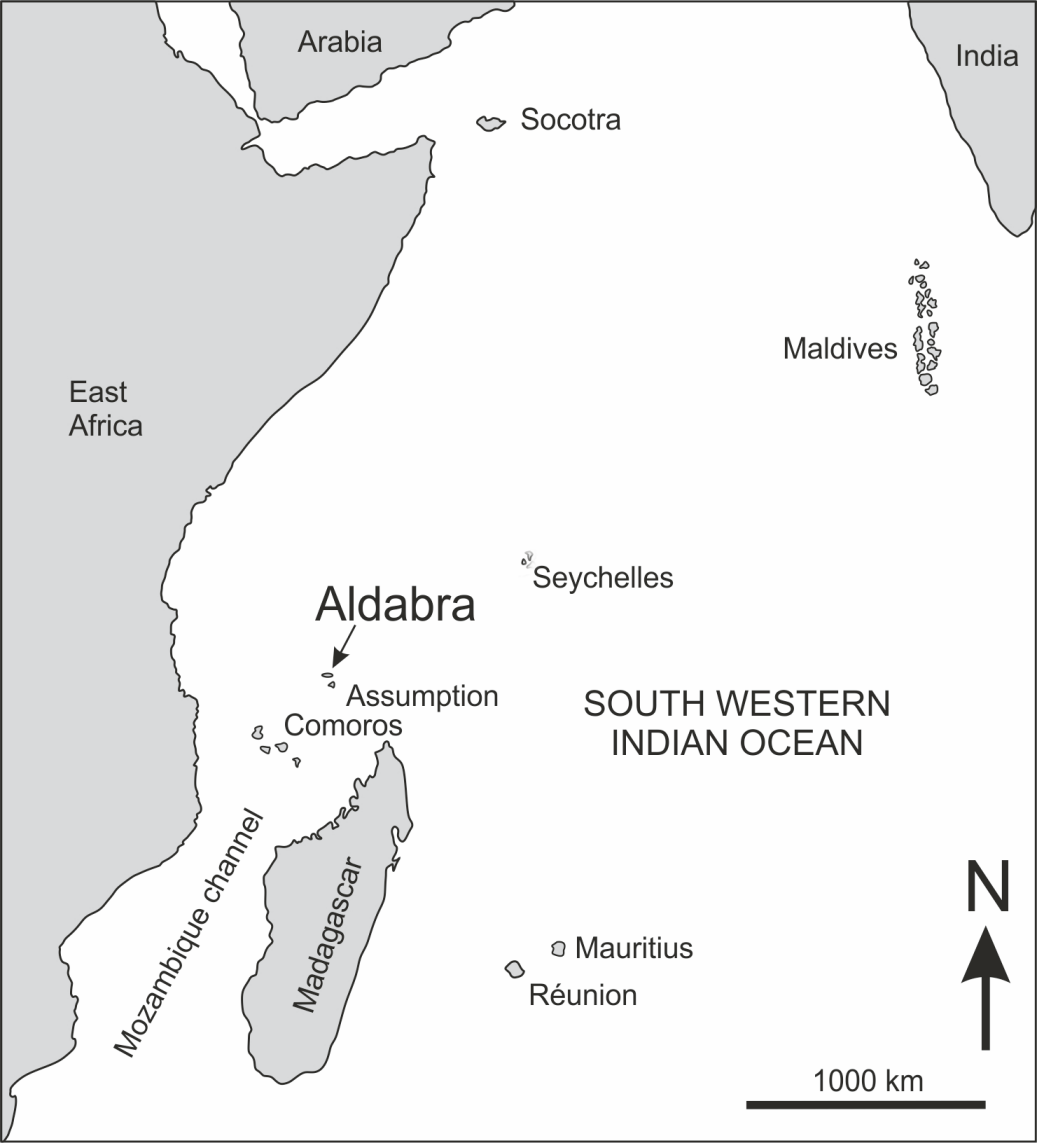
Outline map of the south western Indian Ocean, indicating the location of Aldabra Atoll at the northern end of the Mozambique channel, some 425 kilometres from the northern Madagascan coastline and 630 kilometres from East Africa at the Tanzanian/Mozambique frontier.

**Fig 2:**
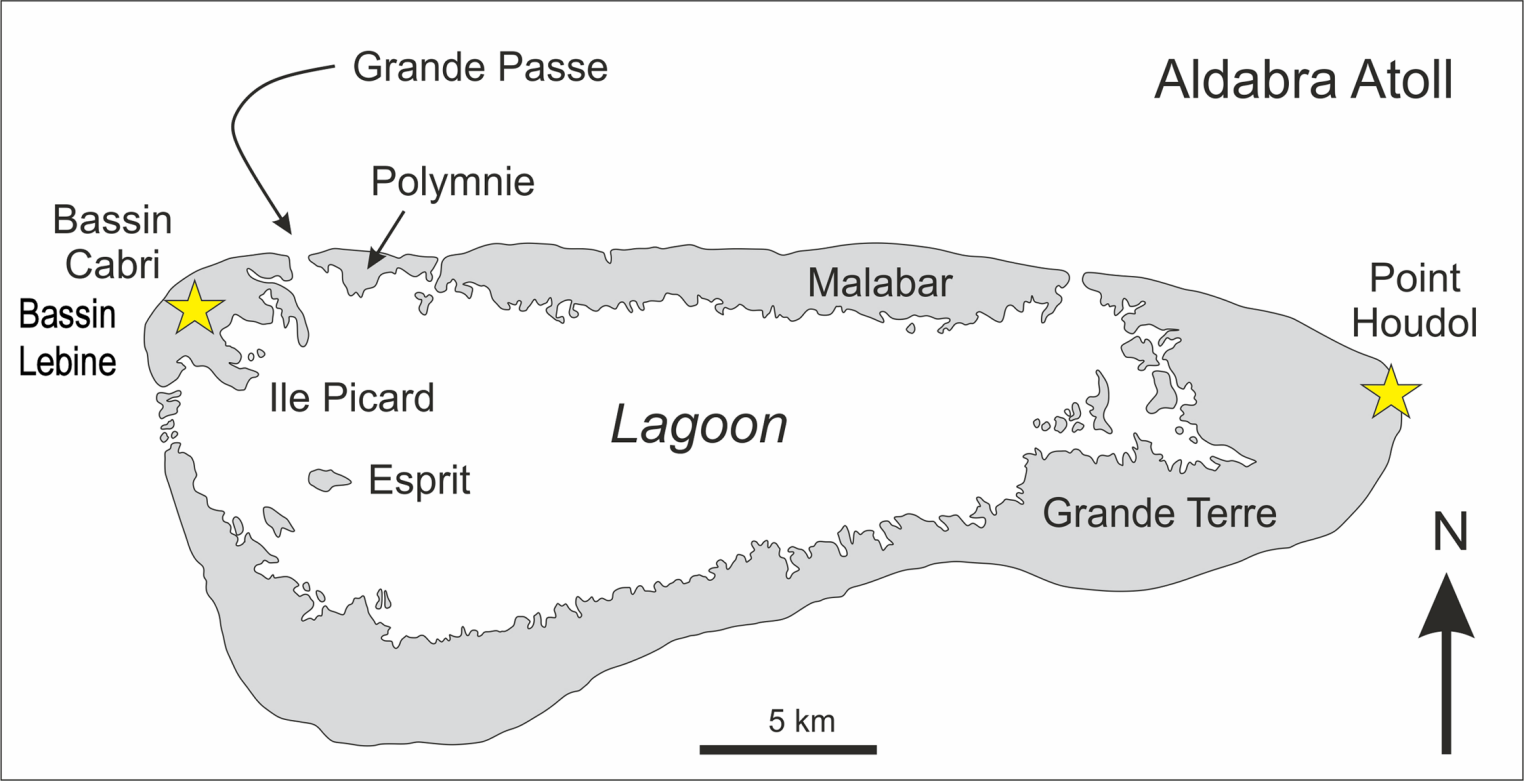
Outline map of Aldabra Atoll indicating the fossil localities discussed in the text.

Aldabra Atoll in the southwestern Indian Ocean is an isolated, raised atoll, the second largest in the world after Kiribati in the central Pacific Ocean (Figs 1 and 2). Aldabra Atoll is famous for harbouring the densest population of giant tortoises anywhere (>100,000), and is also home to the last surviving flightless bird in the Indian Ocean, the endemic subspecies of the White-throated Rail *Dryolimnas cuvieri aldabranus* [1–3]. Because of its unique fauna and flora, including over 400 endemic species, Aldabra was designated the first UNESCO World Heritage Site in 1982 [4]. However, the previous introduction of domestic goats *Capra aegagrus*, domestic cats *Felis catus* and the Black Rat *Rattus rattus* resulted in the decline and probable extinction of some terrestrial gastropods and extinction of the endemic Aldabra Brush Warbler *Nesillas aldabrana*, last recorded in 1983 [5–6].

**Fig 3:**
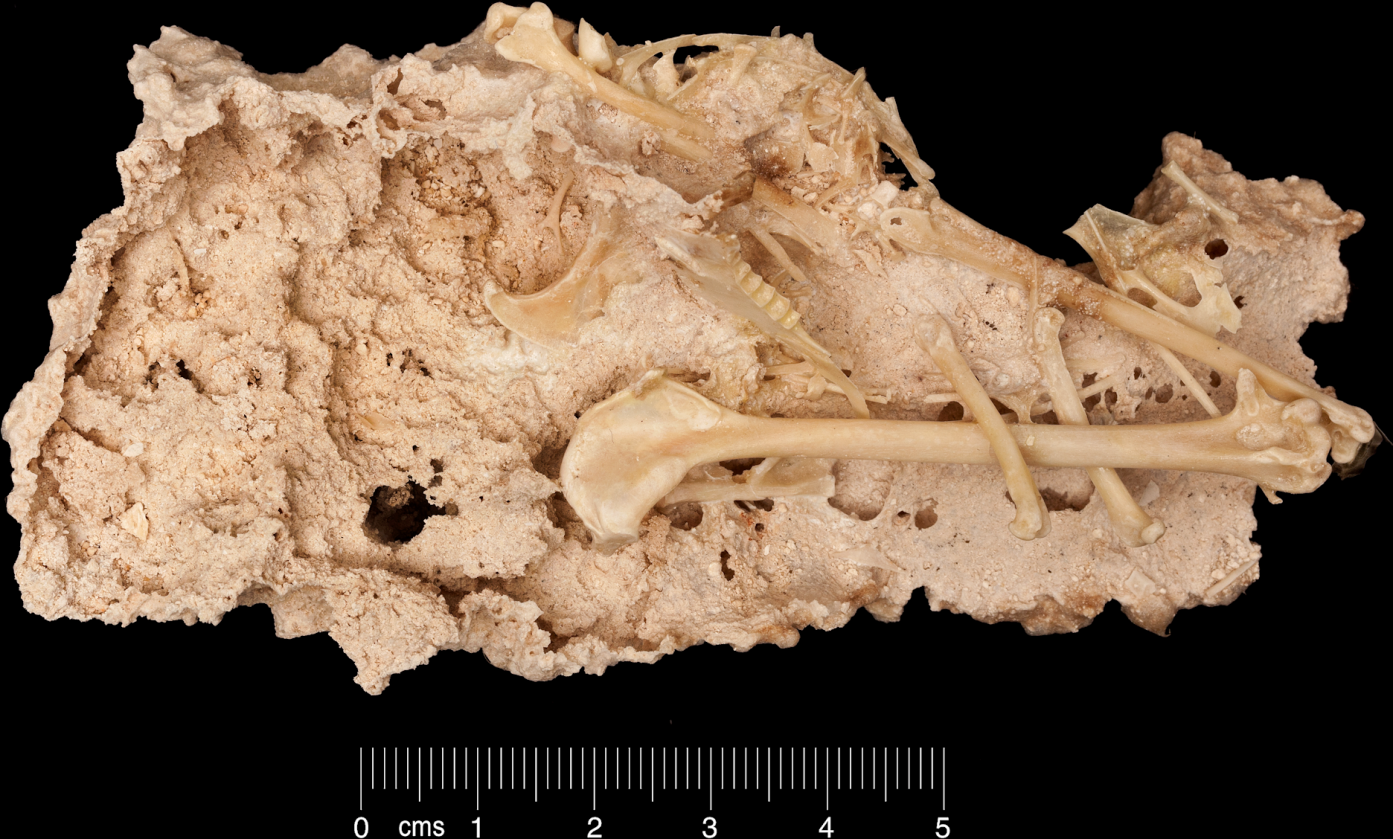
An associated *Pterodroma kurodai* in matrix from the Ile Picard limestone of Bassin Cabri, Ile Picard. Specimen number USNM 78. Scale bar = 10mm.

The atoll includes a number of Pleistocene fossil localities that contain terrestrial gastropod assemblages [7], but only three vertebrate fossil localities are known. These are Bassin Cabri and Bassin Lebine on Ile Picard (West Island) (Fig 2), with deposits older than 136,000 YBP, based on ^230^Th/^234^U dating of the Aldabra Limestone [8–9], and Point Hodoul on Malabar (South Island), the youngest of the three at 100,000 YBP [10]. Limited numbers of fossil reptile remains have been described from Bassin Cabri [11], and Harrison & Walker [9] described an endemic gadfly petrel *Pterodroma kurodai* (Fig 3) and a terrestrial duck *Aldabranus cabri* from the same locality. Conversely, large numbers of reptile bones, including six species of lizard, giant tortoises and a horned crocodilian, *Aldabrachampsus dilophus*, were found at Point Hodoul, but a single bone of *Dryolimnas cuvieri* was the only avian element recovered [9, 11–12]. The material described in this analysis comes from the Bassin Cabri and Bassin Lebine localities.

From March 26 –April 15, 1987, John Becker, formerly of the Smithsonian Institution, collected a large series of fossils encased in matrix from Bassin Cabri and Bassin Lebine, of which many were never fully prepared and none described. Here we present the results of a long-term project to acid-prepare the remaining material collected by Becker, and provide hitherto unrecorded information about the original avian diversity of Aldabra Atoll during the Upper Pleistocene. Furthermore, we examined the holotype and paratype *Pterodroma kurodai* material as well as the *Dryolimas cuvieri* material examined by Harrison & Walker [9], which provide additional details about the faunal diversity of the Atoll.

## Geography

Aldabra Atoll (9°24′S, 46°22′E) is part of the Aldabra Group, comprising Aldabra Atoll, Assumption Island and the atolls of Astove and Cosmeledo. It is situated 425 km to the northwest of Madagascar and belongs to the outer islands of the Seychelles, situated 1,138 km from the granitic islands to the northeast (Fig 1). Aldabra is the second largest raised coral reef in the world with an elevation of 8 m at its highest point [13]. The atoll is surrounded by a coral reef and is 34 km long and 13 km wide with a surface area of 380 km^2^, of which 196 km^2^ comprises a large shallow internal lagoon [13–15]. The lagoon has a very high tidal range for an oceanic atoll, with a mean spring-tide range of 2.74 m [16], with four main tidal passages that connect the lagoon to the surrounding ocean; the largest, Grande Passe (Main Channel), is a 65 m wide and around 20 m deep channel on the north west coast [15–16]. These tidal channels divide Aldabra into four main islands: Grande Terre (South Island), Malabar (Middle Island), Polymnie Island and Ile Picard (West Island) (Fig 2).

Aldabra is subject to the northwest monsoon from *circa* November to March (Austral summer) and south-easterly trade winds which are drier, for the remainder of the year, [17–18]. Aldabra is rarely affected by cyclones, unlike other southwestern Indian Ocean islands (e.g. Mascarene Islands), and receives an average of 960 millimetres (38 in) rainfall per year [18–19]. The highest monthly mean maximum temperature is recorded in December at 31°C (88°F), whereas the lowest mean minimum temperature is recorded in August at 22°C (72°F) [17,18–21].

## Geology and geological history

The surface geology of Aldabra Atoll is represented entirely by Pleistocene and Holocene strata; no data are available for earlier geological events. It is complex, reflecting numerous periods of uplift and submergence, summarised in detail in references [8, 10, 13–14]. There are at least three major marine transgressive events represented entirely by carbonate and phosphate deposition. As there is only one dated sequence (Aldabra Limestone), the age of the others is based on their depositional sequence, and are here presented oldest first.

### The Esprit limestone and phosphorites

The earliest deposits on Aldabra are the Esprit limestone (quiet-water calcarenites) and the Esprit phosphorites of the lagoonal Esprit Island and adjacent area (Fig 2). These were deposited in calm water conditions in a shallow lagoon, at which time the atoll was completely submerged. These deposits contain abundant marine molluscs but very few corals [10] and are older than 136,000 YBP [14]. Lower sea levels saw the exposure of the sediments in sub-aerial conditions, resulting in the cementation and dissolution of the limestone to form a dissected karst topography, at which time phosphates, probably derived from sea bird guano, accumulated in open cavities. At this time Aldabra may have been as large as 365 km^2^, the total area of the bank exposed during the lowest sea levels [10, 14].

### Picard calcarenites

Subsequently, rising waters resulted in the deposition of the still undated Picard Calcarenites that gave rise to an island, which may have been 20 km^2^ in area [10], and was colonised by giant Fs, birds, crocodiles and terrestrial snails (Bassin Cabri and Bassin Lebine fossil deposits). There is also evidence of seasonal bodies of fresh water [10]. During this time extensive sub-aerial disolution and local deposition of terrestrial soils occurred [8].

### Takamaka limestone

The second major marine incursion is represented by the Takamaka Limestone, consisting largely of a fine calcarenite deposited in quiet-water conditions, and characterized by abundant calcareous, benthic red algae, with few corals or molluscs [10, 13]; exposures of this limestone rise to approximately 2 m above present sea level (PSL). The geology suggests that the atoll was possibly totally submerged during deposition of this unit, but the rise of only 2 m above PSL, compared with 8 m during deposition of the Aldabra Limestone (see below), leaves room for doubt; smaller refugia islands may have remained above-sea level.

### Aldabra limestone

A third major marine incursion is represented by the Aldabra Limestone, and all available geological and palaeontological evidence supports a complete inundation event during this time. Deposited between 118,000 and 136,000 YBP ± 9,000, it covers most of the outer rim of the atoll and rises to around 8 m above PSL, forming some low cliffs [13]; the sea level had risen to ~10 m above PSL. It consists of coarse calcarenites and calcirudites with abundant well preserved corals and marine molluscs [8, 14]. A subsequent interrupted fall in sea level, eventually to ~120 m at the last glacial maximum, cut two distinct terraces of 8 m and 4–6 m above PSL along the coast line [13–14]. This event corresponds with geological evidence on Bermuda, South Atlantic, when the onset of the last interglacial (Marine Isotope Stage 5 (MIS 5)) completely inundated the island [22]. Due to falling sea levels, Aldabra now comprised a ring of narrow, low rocky islets surrounding a wide shallow lagoon [10]. Terrestrial deposits (soils) accumulated in the irregular cavities and in pools, and it is these that contain high densities of vertebrate remains [13]; the undated reptile-rich Point Hodoul, a cavity fill deposit, accumulated at some time during this period [10].

### Post 118,000 YBP

Aldabra became a steep-sided, large rocky island, possibly up to 100 m high relative to sea level and perhaps of around 400 km^2^, for an unknown duration during the Würm Glaciation beginning 115,000 YBP, and to the Late Wisconsin (85,000–11,000 YBP) in which the last glacial maximum occurred approximately 25,000–21,000 YBP [10, 13, 23]. The subsequent ocean rise to its PSL, and heavy erosion due to high rainfall, saw the breaching of the land rim by sea water, resulting in a reduction of land surface area by nearly 60% [7, 10]. This event may have occurred as recently as 5,000–7,000 YBP [24–25]. All limestone on Aldabra is heavily eroded by intense dissolution and probable bioerosion, with a karstic topography of jagged pinnacles (*champignon*), flat areas of a probable old lagoon floor (*platin*), and relatively smooth areas broken by deep pot holes and pits (*pavé*) [26]. Eroded reef-platforms have produced fine carbonate sands on the lagoon floor and formed pocket beaches and sand dunes, especially on the windward south coast.

## Petrography and mineralogy of the fossiliferous limestone

A number of studies provided a petrographic study of the carbonates of Aldabra have concluded that the petrology was generally heterogeneous with considerable lateral and vertical facies diversity; mineralogy and porosity were also extremely diverse [26–28]. The limestone has been subject to much reworking, so that large volumes consist of phosphatic cement sequences with infilled cavities or are internal sediments [26]. The analysed samples were simple bioclastic deposits, cemented by high magnesium calcite or aragonite, with the presence of small amounts of phosphates. The petrology of the Picard calcarenites (calcarenite and calcilutite) contained a mineralogy of aragonite, calcite, low and high magnesium calcite and iron oxides [27]. No information is available for Point Hodoul.

## Upper Pleistocene vegetation

The Bassin Cabri and Bassin Lebine area on Ile Picard at the time of the deposition of the Picard calcarenites was probably well vegetated, but with a moist, low, grassy habitat and an absence of true soil development [7]; a terrestrial gastropod fauna was abundant. Fossil pollen and spores reveal a vegetational change occurred at the time of the Takamaka deposits, showing that the atoll had an irregular rocky surface with true soil accumulation in pockets. The abundant and diverse gastropod assemblage suggests open, low vegetation habitats subject to periodic flooding, open wood and scrub with leaf litter development, to drier habitats with open scrub [7]. The diversity of vertebrates and terrestrial gastropods of Point Hodoul’s pipe-fill deposits indicate that the atoll was probably well vegetated, with a complex open or dense scrub and scrub forest, generally xerophytic, and broken by open grass areas, perhaps similar to the present-day vegetation, but with a higher rainfall [7, 29].

## Present-day vegetation

Around 273 species of plants, 19 of which are endemic, have been recorded on Aldabra [29–30]. The vegetation has been divided into zones, with the most important being: Pemphis scrub (*Pemphis acidula*), which forms almost pure stands up to 6 m tall at lower levels and mixes with *Sideroxylon* and other scrub species at higher levels; Mixed scrub and scrub woodland which consists of a large diversity of evergreen species, especially *Pandanus*; and Mangroves that occur within the lagoon and occupy a total area of around 800 ha [30–32]. However, due to absence of terrestrial sediment and considerable flushing due to high tidal ranges, the tallest mangrove trees attain a height of only 10 m, and are generally much shorter [32]. Other vegetation components are: Broadleaf forest comprising *Pisonia*, *Thespesia* and *Calophyllum* (Takamaka); Scaevola coastal scrub of coastal and inland areas; and *Sporobolus* grassland, *Sclerodactylon* tussock grassland and Tortoise turf which are heavily grazed by giant tortoises. Tortoise turf is a distinct association of grasses, sedges and herbs [2, 30]. Because they occur in the areas of the highest tortoise concentration and are subject to intense tortoise grazing, more than half of these Tortoise turf species are genetically dwarfed and consequently has evolved highly specialised growth strategies, e.g. flowers and fruits produced at the base of the plants [31].

## Present day vertebrate fauna

The avian vertebrate fauna comprises 12 endemic terrestrial birds and 14 breeding natives [3, 33–34] (Table 1), and numerous migrant and vagrant species, notably waders, occur, as comprehensively listed by Safford & Hawkins [3] and SRBC [34]. No native terrestrial mammals occur, but five bats (three endemic) are present [35–37]. Reptiles make up the remaining vertebrate fauna, which consists of the largest and densest population of giant tortoises, *Aldabrachelys gigantea* anywhere [2], but unlike in the Pleistocene assemblage, when Aldabran lizard diversity was high, only three species (one endemic) now inhabit the atoll [38–39] (Table 2).

**Table 1:** List of endemic, indigenous breeding and vagrant bird species that occur or occurred on Aldabra, compared with related subspecies on Madagascar and the Comoros, the likely source areas [See 11–12, 37–38].

Aldabra	Upper Pleistocene (Ile Picard)	Upper Pleistocene (Point Hodoul)	Madagascar	Comoros
† End. Aldabra Petrel *Pterodroma kurodai*	Present	No record		
Large *Pterodroma*	Present	No record		
*Puffinus* sp.	Present	No record		
Ind. Tropical Shearwater *Puffinus bailloni nicolae*	No record	No record		Tropical Shearwater *Puffinus bailloni temptator*
Undescribed storm petrel *Oceanodroma* cf. *monorhis*	Present	No record	Vag. Swinhoe’s Storm Petrel *Oceanodroma monorhis*	Vag. Swinhoe’s Storm Petrel *Oceanodroma monorhis*
Ind. Red-tailed Tropicbird *Phaethon rubricauda rubricauda*	Present	No record	Ind. Red-tailed Tropicbird *Phaethon rubricauda rubricauda*	Ind. Red-tailed Tropicbird *Phaethon rubricauda rubricauda*
Ind. White-tailed Tropic Bird *Phaethon lepturus*	No record	No record	Ind. White-tailed Tropic Bird *Phaethon lepturus*	Ind. White-tailed Tropic Bird *Phaethon lepturus*
Ind. Red-footed Booby *Sula sula rubripes*	No record	No record	Ind. Red-footed Booby *Sula sula rubripes*	Ind. Red-footed Booby *Sula sula rubripes*
Ind. Lesser Frigatebird *Fregata ariel iredalei*	No record	No record	Ind. Lesser Frigatebird *Fregata ariel iredalei*	Ind. Lesser Frigatebird *Fregata ariel iredalei*
Ind. Great Frigatebird *Fregata minor aldabrensis*	No record	No record		Ind. Great Frigatebird *Fregata minor aldabrensis*
Ind. Madagascar Pond Heron *Ardeola idae*	Present?	No record	Ind. Madagascar Pond Heron *Ardeola idae*	Ind. Madagascar Pond Heron *Ardeola idae*
Ind. Grey Heron *Ardea cinerea*	No record	No record	Ind. Grey Heron *Ardea cinerea*	Ind. Grey Heron *Ardea cinerea*
Ind. Dimorphic Egret *Egretta* (*garzetta*) *dimorpha*.	No record	No record	Ind. Dimorphic Egret *Egretta* (*garzetta*) *dimorpha*.	Ind. Dimorphic Egret *Egretta* (*garzetta*) *dimorpha*.
End. Aldabra Sacred Ibis *Threskiornis bernieri abbotti*	No record	No record	Ind. Sacred Ibis *Threskiornis bernieri bernieri*	
† End. Aldabra Duck *Aldabranus cabri*	Present	No record		
Undescribed harrier *Circus* sp.	Present	Present	Ind. Madagascar Harrier *Circus macrosceles*	Ind. Madagascar Harrier *Circus macrosceles*
End. Aldabra Kestrel *Falco newtoni aldabranus*	No record	No record	Ind. Madagascar Kestrel *Falco newtoni newtoni*	Vag. Madagascar Kestrel *Falco newtoni newtoni*
End. Aldabra White-throated Rail *Dryolimnas cuvieri aldabranus*	Present	White-throated Rail *Dryolimnas cuvieri* cf. *cuvieri*	Ind. White-throated Rail *Dryolimnas cuvieri cuvieri*	Ind. White-throated Rail *Dryolimnas cuvieri cuvieri*
Undescribed gull *Larus* sp.	Present	No record		
Ind. Swift Tern *Thalasseus bergii thalassinus*	No record	No record	?	
Ind. Black-naped Tern *Sterna sumatrana mathewsi*	No record	No record		
Ind. Caspian Tern *Hydroprogne caspia*	No record	No record	Ind. Caspian Tern *Hydroprogne caspia*	Ind. Caspian Tern *Hydroprogne caspia*
Ind. Fairy Tern *Gygis alba*	No record	No record	Ind. Fairy Tern *Gygis alba*	Ind. Fairy Tern *Gygis alba*
Ind. Brown Noddy *Anous stolidus pileatus*	No record	No record	Ind. Brown Noddy *Anous stolidus pileatus*	Ind. Brown Noddy *Anous stolidus pileatus*
End. Aldabra Turtle Dove *Nesoenas picturata coppingeri*	No record	No record	Ind. Madagascar Turtle Dove *Nesoenas picturata picturata*	End. Comoros Turtle Dove *Nesoenas picturata comorensis*
End. Aldabra Blue Pigeon *Alectroenas sganzini minor*	No record	No record	End. Madagascar Blue Pigeon *Alectroenas madagascariensis*	End. Comoro Blue Pigeon *Alectroenas sganzini sganzini*
† Ind. Barn Owl *Tyto alba*	Present	Present (inference)	Ind. Barn Owl *Tyto alba*	Ind. Barn Owl *Tyto alba*
End. Aldabra Coucal *Centropus toulou insularis*	No record	No record	End. Madagascar Coucal *Centropus toulou toulou*	
End. Aldabra Nightjar *Caprimulgus madagascariensis aldabrensis*	No record	No record	End. Madagascar Nightjar *Caprimulgus madagascariensis madagascariensis*	
End. Aldabra Bulbul *Hypsipetes madagascariensis rostratus*	No record	No record	Ind. Madagascar Bulbul *Hypsipetes madagascariensis madagascariensis*	Ind. Madagascar Bulbul *Hypsipetes madagascariensis madagascariensis*
End. Aldabra Drongo *Dicrurus aldabranus*	No record	No record	End. Madagascar Crested Drongo *Dicrurus forficatus forficatus*	End. Comoro Crested Drongo *Dicrurus forficatus potior*
† End. Aldabra Brush Warbler *Nesillas aldabrana*	No record	No record	End. Subdesert Brush Warbler *Nesillas lantzii*/Malagasy Brush Warbler *Nesillas typica*	End. Grand Comoro Brush Warbler *Nesillas brevicaudata*/Anjouan Brush Warbler *Nesillas longicaudata*/Moheli Brush Warbler *Nesillas mariae*
End. Souimanga Sunbird *Nectarina souimanga aldabrensis*	No record	No record	End. Madagascar Sunbird *Nectarina souimanga souimanga*/South *C*. *s*. *apolis*	
End. Aldabra White-eye *Zosterops maderaspatana aldabrensis*	No record	No record	End. Madagascar White-eye *Zosterops maderaspatana maderaspatana*	End. Moheli White-eye *Zosterops maderaspatana comorensis*
End. Aldabra Fody *Foudia aldabrana*	No record	No record	End. Madagascar Fody *Foudia madagascariensis*/Forest Fody *Foudia omissa*	End. Red-headed Fody *Foudia eminentissima*

Key:

† = Extinct

End = Endemic; Ind = Indigenous; Vag = Vagrant

? = Uncertain.

**Table 2:** List of endemic and indigenous breeding reptile species that occur or occurred on Aldabra, compared with related taxa on Madagascar and the Comoros, the likely source areas.

Aldabra	Upper Pleistocene (Ile Picard)	Upper Pleistocene (Point Hodoul)	Madagascar	Comoros
End. † Aldabra Horned Crocodile *Aldabrachampsus dilophus*	No record	Present		
End. Aldabra Giant Tortoise *Aldabrachelys gigantea*	Present	Present	End. † Malagasy Giant Tortoise *Aldabrachelys abrupta*	
End. Aldabra Snake-eyed Skink *Cryptoblepharus aldabrae*	No record	No record	Ind. Madagascar Snake-eyed Skink *Cryptoblepharus boutonii*	Ind. Madagascar Snake-eyed Skink *Cryptoblepharus boutonii*
End?. † Speckle-lipped Skink ?*Trachylepis* aff. *comorensis*	Present	Present		End. Speckle-lipped Skink *Trachylepis comorensis*
End?. † Anjouan Skink *Amphiglossus* cf. *johannae*	No record	Present	End. Malagasy *Amphiglossus* (21 species)	End. Anjouan Skink *Amphiglossus johannae*
Ind. Leaf-toed Gecko *Hemidactylus mercatorius*	No record	No record	Ind. Leaf-toed Gecko *Hemidactylus mercatorius*	Ind. Leaf-toed Gecko *Hemidactylus mercatorius*
Ind. † Ground Gecko *Paroedura* cf. *sanctijohannis* or *Paroedura* cf. *stumpffi*	No record	Present	Ind. Madagascan Ground Gecko *Paroedura stumpffi*	Ind. Comoro Ground Gecko *Paroedura sanctijohannis*
Ind. † Peter’s Spotted Gecko *Geckolepis* cf. *maculata*	No record	Present	Ind. Peter’s Spotted Gecko *Geckolepis maculata*	Ind. Peter’s Spotted Gecko *Geckolepis maculata*
End. Abbott’s Day Gecko *Phelsuma abbotti abbotti*	No record	No record	End. Abbott’s Day Gecko *Phelsuma abbotti chekei*	
Ind? † Day Gecko *Phelsuma* sp.	No record	Present	?	?
End? † Collared Iguana *Oplurus* cf. *cuvieri*	Present	Present	Ind. Madagascan Collared Iguana *Oplurus cuvieri cuvieri*	End. Comoro Collared Iguana *Oplurus cuvieri comorensis*

Key:

† = Extinct

End = Endemic; Ind = Indigenous

? = Uncertain.

## Introduced fauna

A number of alien species have been introduced to Aldabra Atoll since its settlement by humans in 1880, which include the accidental introduction of black rat *Rattus rattus* (*circa* 1890) and some lizards, and the intentional introduction of goat *Capra aegagrus* (*circa* 1878) and cat *Felis catus* (*circa* 1892) [40]. The mammalian introductions have been particularly detrimental to the endemic fauna of Aldabra.

## Materials and methods

All fossil material mentioned is accessioned to the collection of the United States National Museum (USNM) except where stated otherwise. A large amount of fossil material had been prepared prior to this study, with the remaining fossil samples still in matrix. These were submerged in 10% acetic acid to free the fossiliferous material, and once this was completed the acetic acid was neutralised in water. No additional preparation was undertaken on the majority of the specimens, but the more delicate specimens were coated with Paraloid B-72 to consolidate them and prevent damage. Fossil material was compared with Aldabra fossil specimens, held at the Palaeontology division of the Department of Earth Sciences, Natural History Museum, London (NHMUK), which were collected in 1969 by Dr. J. D. Taylor. All measurements were made using a dial calipers and rounded to the nearest 0.1 mm. Much of the fossil material used in this analysis is fragmentary, so obtaining measurements was sometimes problematic. Osteological abbreviations used in the text are R (right), L (left), p (proximal) and d (distal). Bird nomenclature follows Safford & Hawkins [3]. Unless otherwise stated, all fossil material is bulk numbered USNM 381095.

## Results

Systematic Palaeontology.

Aves.

Procellariformes Fürbringer, 1888 [41].

Procellariidae Leach, 1820 [42].

*Pterodroma* Bonaparte, 1856 [43].

*Pterodroma kurodai* Harrison & Walker, 1978 [9].

Material: 380+ bones representing nearly all parts of the cranial and post cranial skeleton.

Fossil locality: Bassin Cabri and Bassin Lebine.

Status: Extinct, endemic, known only from fossils.

**Fig 4:**
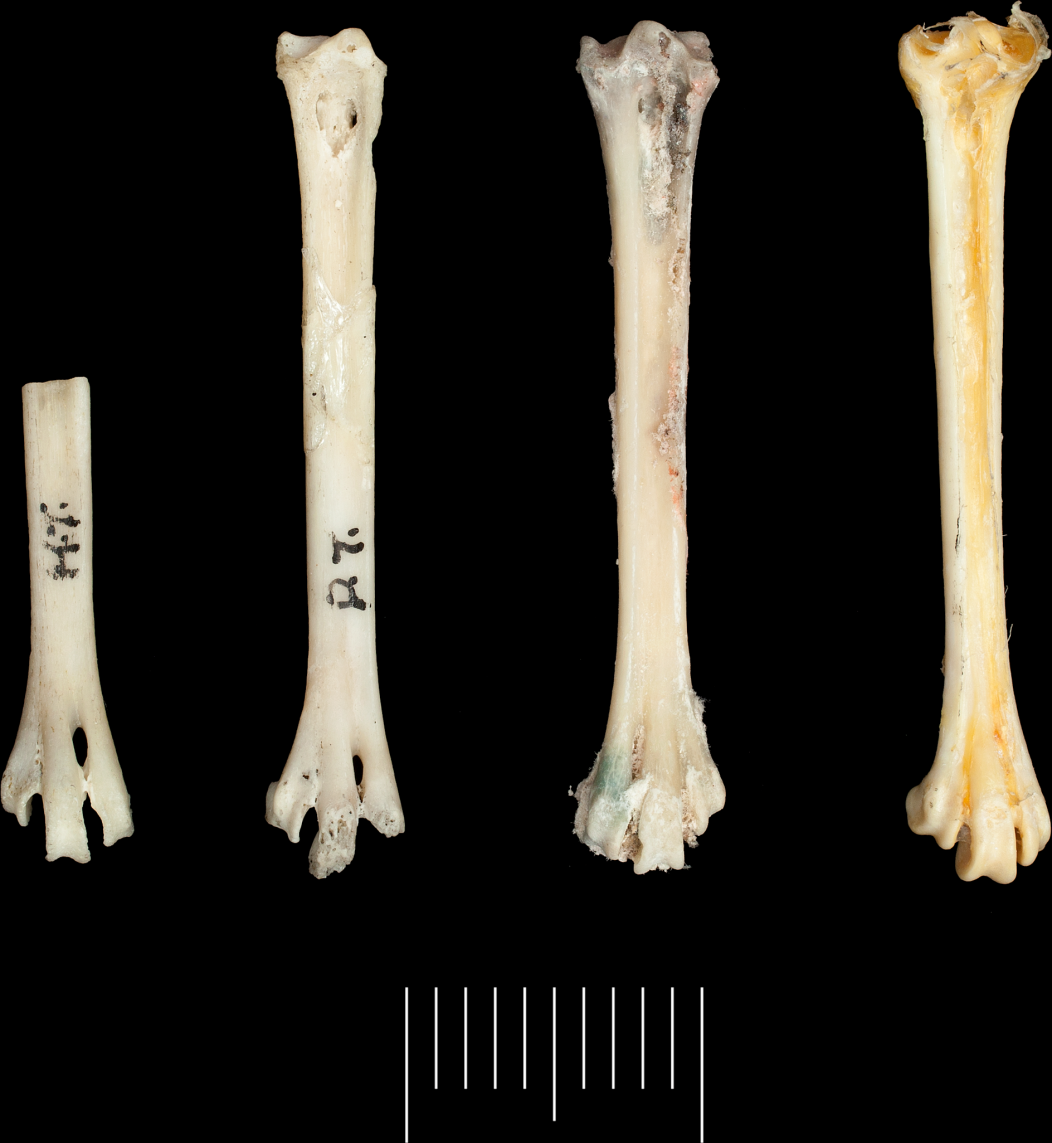
Tarsometatarsi (left side) of *Pterodroma kurodai* Harrison & Walker, 1978 from Bassin Cabri, Ile Picard, compared with new material of *P*. *kurodai* from Ile Picard and *P*. *leucoptera*; From left to right, holotype NHMUK A4361; NHMUK A4632; USNM 78; *P*. *leucoptera* NHMUK S/1974.12.1. Scale bar = 10mm.

Remarks: From the material collected by Becker, including specimens already prepared by him, we have 416 identifiable avian bones, of which 362 are specifically assigned to *Pterodroma kurodai*, i.e. 87%. The deposit includes articulated specimens and juveniles, which confirms that a breeding population was present at Bassin Cabri and Bassin Lebine. *P*. *kurodai* is slightly larger in post-cranial elements than the otherwise similar-sized *P*. *leucoptera* (Fig 4), but smaller than any extant *Pterodroma* petrels from the south-western Indian Ocean, and Mascarene Petrel *Pseudobulweria aterrima* from Réunion Island, Mascarenes.

*Pterodroma* sp. (large).

Material: Ld, Rd and Rp humerus; R humerus shaft fragment; Ld and Rd ulna; Ld carpometacarpus; R femur; Lp, Ld tibiotarsus.

Fossil locality: Bassin Lebine.

Status: Extant?

Remarks: Unassociated premaxilla and post-cranial bones belonging to a large *Pterodroma* species much larger than *P*. *kurodai* show that a second species was present on Aldabra in the Upper Pleistocene. Given the scarcity of remains, this taxon may not have been a resident breeder.It could represent one of the extant larger *Pterodroma* species present today elsewhere in the Indian Ocean, but more material is needed to confirm this. Species that fall within the size range and inhabiting the Indian Ocean are Trindade Petrel *P*. *arminjoniana* and Kermadec Petrel *P*. *neglecta*.

*Puffinus* Brisson, 1760 [44].

*Puffinus* sp.

Material: Associated and partially articulated post-cranial skeleton, plus premaxilla; R humerus shaft; Rd femur; Ld tibiotarsus; R, L, Lp, Ld, Rd, Rp tarsometatarsus.

Fossil locality: Bassin Cabri and Bassin Lebine.

Status: Extinct/extirpated?

Remarks: The Tropical Shearwater *Puffinus bailloni* is the only resident breeding species of procellarid on Aldabra, whereas the osteologically similar Wedge-tailed Shearwater *Puffinus pacifica* and Flesh-footed Shearwater *P*. *carneipes* are occasional vagrants to Aldabra. The procellarid fossils are too small to belong to *P*. *carneipes*, are too large to belong to *P*. *bailloni*, but are within the smaller size range of *P*. *pacificus*, which is common throughout the Indian Ocean and breeds on many islands [3]; the fossils are approximately the same size as Manx Shearwater *P*. *puffinus*, although the latter does not occur in the Indian Ocean region. The small size range of the fossil material suggests that a different species of *Puffinus* may once have occurred on Aldabra, but is no longer present.

Hydrobatidae Mathews, 1912 [45].

*Oceanodroma monorhis*? (Swinhoe, 1867) [46].

Material: Rp ulna.

Fossil locality: Bassin Lebine.

Status: Extant?

Remarks: This ulna fragment is the only evidence that a probable *Oceanodroma* storm petrel occurred on Aldabra during the Upper Pleistocene. Swinhoe’s Storm-petrel *Oceanodroma monorhis* is a rare straggler to the atoll [3], and the specimen is within its size range, but too fragmentary to identify to species.

Phaethontiformes Sharpe, 1891 [47].

Phaethontidae Brandt, 1840 [48].

*Phaethon* Linnaeus, 1758 [49].

*Phaethon rubricauda* Boddaert, 1783 [50].

Material: Rd coracoid; L humerus; x2 Ld humerus; Rd humerus; L ulna; Rd carpometacarpus.

Fossil locality: Bassin Cabri and Bassin Lebine.

Status: Extant.

Remarks: The Red-tailed Tropicbird is a common breeding resident on Aldabra today, and is widespread throughout the Indian and Pacific oceans. Although scarce, the fossil remains comprise two individuals which suggests that it was also a resident species in the Upper Pleistocene of Aldabra.

Pelecaniformes Sharpe, 1891 [47].

Ardeidae Leach, 1820 [42].

?*Ardeola* sp. F. Boie, 1822 [51].

Material: Ld femur.

Fossil locality: Bassin Lebine.

Status: Extant/extirpated.

Remarks: This femur, although fragmentary, is almost certainly referable to an *Ardeola* pond heron. The Madagascar Pond Heron *Ardeola idae* breeds in small numbers on Aldabra today, which confirms that it or a similar species was present on Aldabra in the Upper Pleistocene.

Accipitriformes Vieillot, 1816 [52].

Accipitridae Vieillot, 1816 [52].

Circinae Sundevall, 1836 [53].

*Circus* Lacépède 1799 [54].

*Circus* sp.

Material: Associated, but crushed Lp coracoid; L radius shaft fragment; Lp tibiotarsus fragment; L carpometacarpus; Rd femur. Also Rp ulna; L tibiotarsus; Rd tarsometatarsus; ungual (Bassin Lebine).

Fossil locality: Bassin Cabri and Bassin Lebine.

Status: Extant?

Remarks: A least three individuals of a *Circus* sp. are represented in the fossil deposits, which strongly suggests that a local breeding population was present. The fossils are in the size range of the endemic Indian Ocean island species, Réunion Harrier *C*. *maillardi*, which today is known only from Réunion (extant) and Mauritius (extirpated) [55], but is smaller than Madagascar Marsh Harrier *C*. *macrosceles*, which occurs on Madagascar and the Comoros [3]. Two species of *Circus*, Pallid Harrier *C*. *macrourus*, which is of similar size, and Western Marsh Harrier *C*. *aeruginosus*, which is much larger, are rare vagrants to the Seychelles [34], but neither have been recorded on Aldabra.

Gruiformes Bonaparte, 1854 [55].

Rallidae Rafinesque, 1815 [56].

*Dryolimnas* Sharpe, 1893 [57].

*Dryolimnas cuvieri* Pucheran, 1845 [58].

*Dryolimnas cuvieri* cf. *aldabranus* Günther, 1879 [59].

Material: L humerus; Rd humerus (USNM UJP79).

Fossil locality: Bassin Cabri.

Status: Extant.

**Fig 5:**
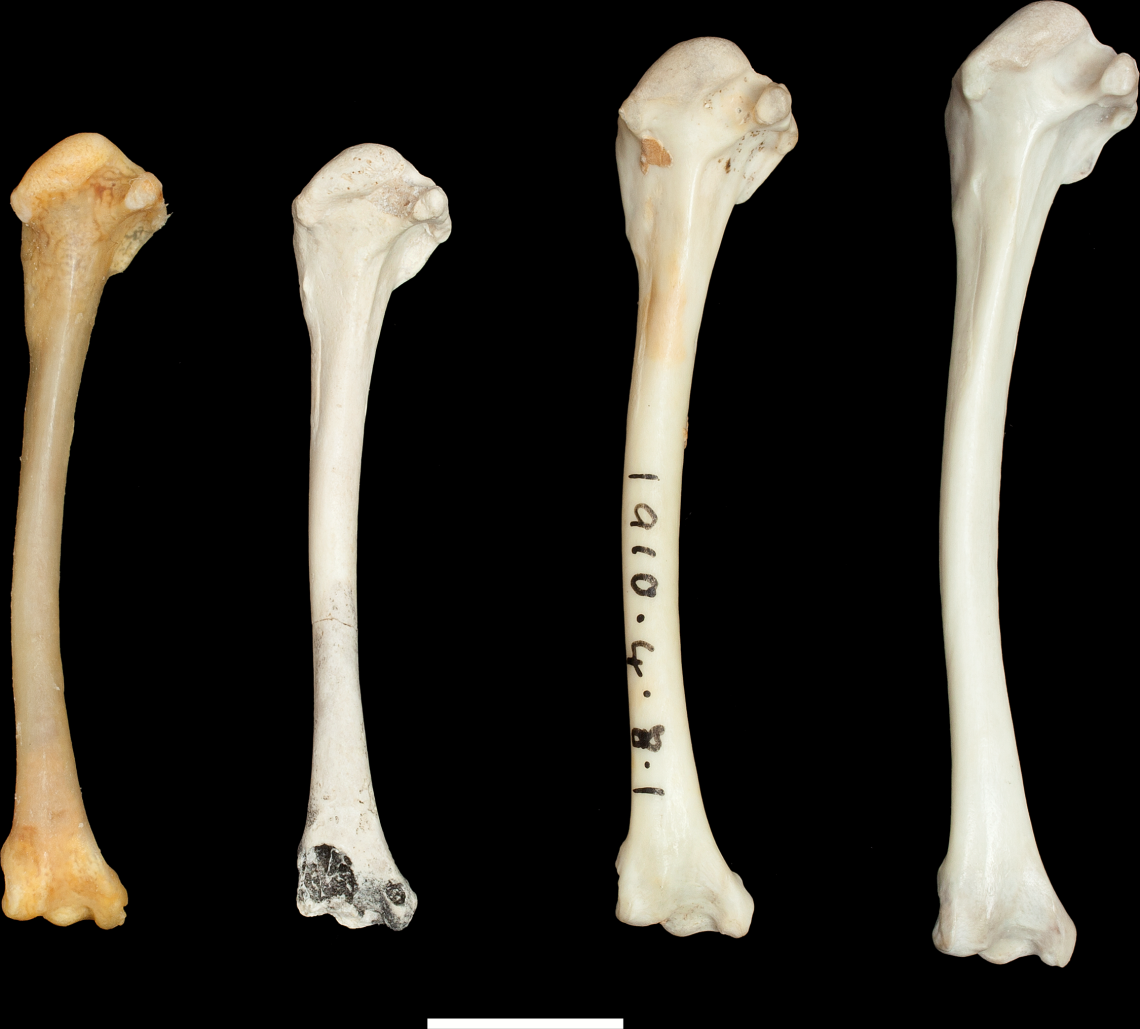
A comparison of humeri (left side) of *Dryolimnas*. From left to right, *D*. *cuvieri aldabranus* NHMUK S/1989.38.7 ♂; *D*. *cuvieri* (Upper Pleistocene) USNM UJP79 unsexed; *D*. *c*. *abbotti* NHMUK 1910.4.8.1 unsexed; *D*. *c*. *cuvieri* NHMUK 1897.5.10.47 unsexed. Scale bar = 10mm.

Remarks: A complete left and distal right humerus of White-throated Rail *Dryolimnas cuvieri* falls within the size range of the present flightless population of *D*. *c*. *aldabranus*, suggesting that the taxon was similarly flightless during the Upper Pleistocene (Fig 5). A distal tarsometatarsus collected from Point Hodoul is more robust than volant *D*. *c*. *cuvieri* and *D*. *c*. *aldabranus*, being more similar in size and morphology to the recently extinct, flightless subspecies, *D*. *c*. *abbotti* from Assumption Island [9].

Charadriiformes Huxley, 1867 [60].

Laridae Rafinesque, 1815 [56].

*Larus* Linnaeus, 1758 [49].

*Larus* sp.

Material: Rd humerus.

Fossil locality: Bassin Lebine.

Status: Extant?

Remarks: A distal end of a humerus confirms the presence of *Larus* on Aldabra during the Upper Pleistocene. The only *Larus* species recorded from Aldabra is Lesser Black-backed Gull *Larus fuscus*, which is a rare vagrant [3], but this species is inseparable osteologically from Yellow-legged Gull *L*. *micahellis*, which may have occurred further south in the past. Two other gull genera, *Chroicocephalus* sp. and Sooty Gull *Icthyaetus hemprichii* are vagrant to the main Seychelles islands, but are too small to be referable. This individual fossil specimen is similar in morphology and size to *L*. *fuscus*, but with only a single bone available, we suggest a similarity to a *Larus* species, rather than assigning it specifically to *L*. *fuscus*.

Strigiformes Wagler, 1830 [61].

Tytonidae Ridgway, 1914 [62].

*Tyto* Billberg, 1828 [63].

*Tyto* sp.

Material: distal ulna.

Fossil locality: Bassin Cabri.

Status: Extinct/extirpated.

**Fig 6:**
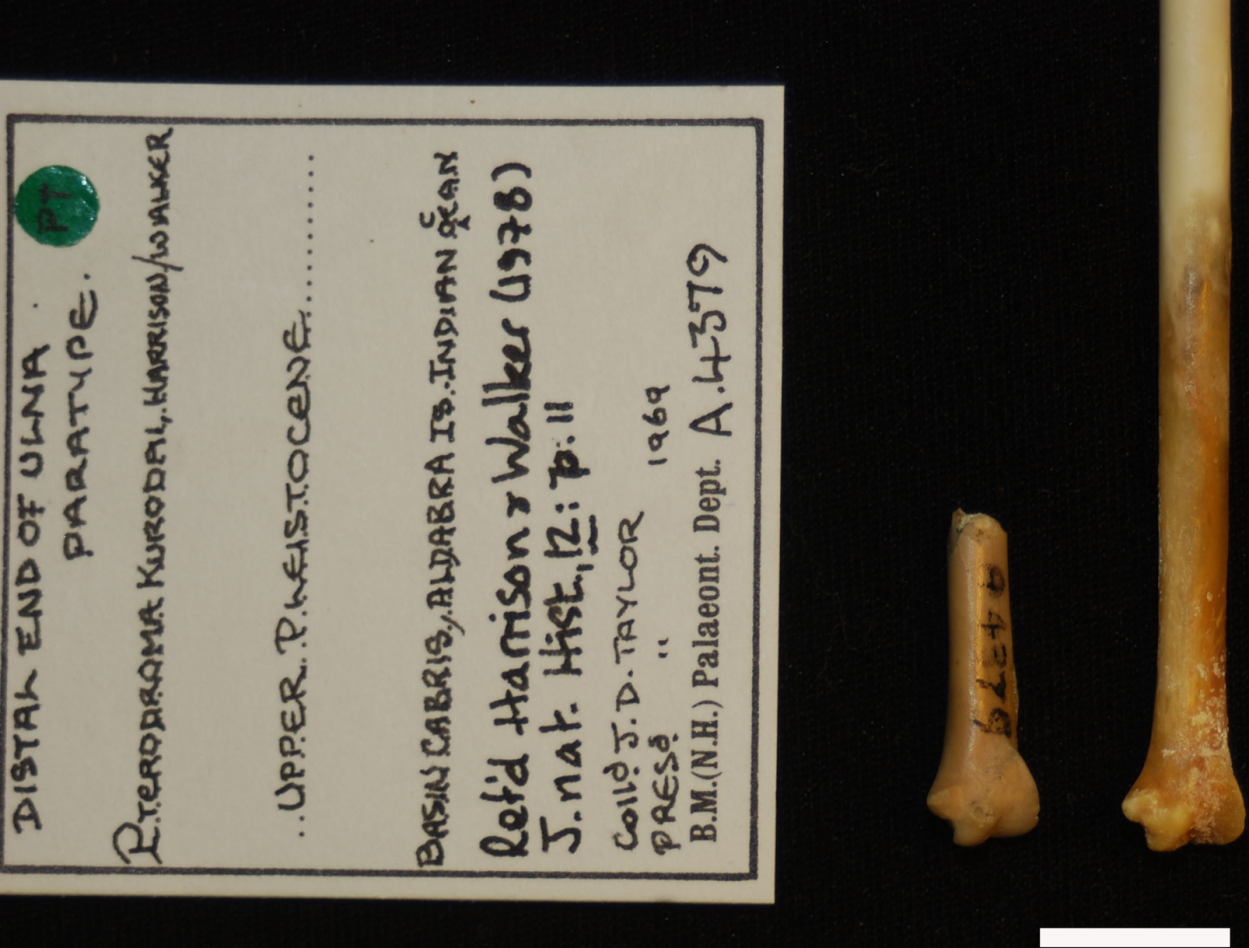
Material erroneously labelled as *Pterodroma kurodai* (as a paratype NHMUK A4379) by Harrison & Walker, 1978, is here referred to the strigiform *Tyto alba* (NHMUK S/2013.1.18 ♀).

Remarks: This specimen NHMUK A4379 was mis-identified by Harrison & Walker [9] as a paratype of *Pterodroma kurodai* (Fig 6). It was not described or listed in their description of that species, so a zoological nomenclature resolution is not necessary here. The specimen is similar in size and morphology to Barn Owl *Tyto alba*, and within the individual size variation exhibited in this species. Because of the fragmentary nature of the specimen, little more can be said about it.

Testudines Batsch, 1788 [64].

Cryptodira Cope, 1868 [65].

Testudinoidea Fitzinger, 1826 [66].

Testunidae Batsch, 1788 [64].

*Aldabrachelys* Loveridge & Williams, 1957 [67].

*Aldabrachelys gigantea* Loveridge & Williams, 1957 [67].

Material: vertebra; scapula-coracoid fragment (USNM 96A); phalanx ungual.

Fossil locality: Bassin Cabri.

Status: Extant.

Remarks: Giant tortoises were present and presumably common on Aldabra during the Upper Pleistocene and remain common today. They appear to be of Madagascan origin, therefore as Aldabra was presumably colonised by the Madagascar Giant Tortoise *Aldabrachelys abrupta*, it has been suggested that it recolonised on at least three occasions after the submergence of the atoll during high sea stands [7]. However, it is equally likely that small areas of land allowed survival of giant tortoises during some of the marine incursions.

Squamata Oppel, 1811 [68].

Lacertilia Günther, 1867 [69].

Scincomorpha Camp, 1923 [70].

Scincidae Gray, 1825 [71].

*Trachylepis* Fitzinger, 1843 [72].

?*Trachylepis* sp.

Material: dentary; vertebrae.

Fossil locality: Bassin Cabri.

Status: Extinct/extirpated, known from fossils only.

Remarks: Arnold [11] described *Mabuya* (now *Trachylepis*) skinks from Point Hodoul, and these larger fossil elements confirm that a *Trachlepis* skink, most similar in size to *T*. *comorensis*, also occurred in the older deposits at Bassin Cabri.

Iguania Gray, 1827 [73].

Opluridae Titus & Frost, 1996 [74].

*Oplurus* cf. *cuvieri* Gray, 1831 [75].

Material: R dentary fragment (USNM UPJ15); L dentary fragment; R humerus; x3 vertebrae.

Fossil Locality: Bassin Cabri.

Status: Extinct/extirpated.

Remarks: Arnold [11] described an *Oplurus* iguana from Point Hodoul, but was unable to confirm if it was conspecific with the Madagascar species, *Oplurus cuvieri*, or represented a distinct taxon. The discovery of *Oplurus* fossil material from Bassin Cabri, the first recorded from the older deposit, confirms that iguanas were present before and after the Ile Picard/Point Hodoul submergence event.

## Discussion

Analysis of the Bassin Cabri and Bassin Lebine fossil localities reveals a more diverse faunal composition in the Upper Pleistocene than previously documented, especially among sea birds. During deposition of the Picard calcarenites, as evidenced at Bassin Cabri, Aldabra at 136,000+ YBP, Aldabra was an extensive, prograding carbonate sand cay that developed behind an active reef, rose to a probable maximum of 2 m above sea level, and was heavily vegetated but lacked true soil development [7, 13]. A probable shallow dune field occurred at the western end, in which standing bodies of fresh water occurred between the dunes [13]. The island was colonised by terrestrial snails, including *Tropidophora*, which is an inhabitant of drier, more open areas, an *Oplurus* iguanid lizard, giant tortoises, an endemic population of gadfly petrels *Pterodroma kurodai*, and a terrestrial duck *Aldabranus cabri* [7, 9, 11–12]. This duck is known from just two fragmentary bones collected at Bassin Cabri, with no new material recovered in this study. Alongside the above-named fauna was alarge *Pterodroma*, a *Puffinus* shearwater, an *Oceanodroma* storm petrel, a flightless White-throated Rail *D*. *cuvieri* and possibly resident/endemic populations of a *Circus* harrier and a *Tyto* barn owl. Sea and shore birds included Red-tailed Tropicbird *P*. *rubricauda*, a *Larus* gull and a possible *Ardeola* pond heron. Giant tortoise *A*. *gigantea* and at least two terrestrial lizards, *Trachylepis* and *Oplurus*, were also present, the last-named were previously only known from Point Hodoul. Crocodilian teeth have been reported from this deposit, and used to be thought to represent Nile Crocodile *Crocodylus niloticus* [11, 39], but all have now been referred to the endemic horned crocodile *Aldabrachampsus dilophus* [12].

The younger deposit of Point Hodoul, at 100,000 YBP as inferred by [14], is separated from the Bassin Cabri deposits by the Aldabra limestone [11]. The Point Hodoul deposit indicates higher precipitation, with resulting denser scrub and leaf litter development [7]. A much greater diversity of fossil reptiles is reported from this deposit (Table 2), including *A*. *dilophus*, *Trachylepis* and *Scelotes* skinks, *Geckolepis*, *Paroedura* and *Phelsuma* gecko species and numerous giant tortoise bones. The only bird is represented by a single distal tarsometatarsus of White-throated Rail *Dryolimnas cuvieri* [9]. The high density of lizards, particularly as they include nocturnal species [11], suggests predation by owls, and although from the older Bassin Cabri deposits, the discovery of a *Tyto* element confirms its presence during the Upper Pleistocene. Furthermore, a Barn Owl *Tyto alba* occurred on Aldabra in recent times until 1906 [33], but it was not formally described and no museum specimen exists.

The complete submergence of the Aldabra platform, evidenced by the Aldabra Limestone unit separating the younger Point Hodoul deposit from that of Bassin Cabri and Bassin Lebine, saw the complete extinction, in the case of the Aldabra Petrel and Aldabra Duck, or extirpation of much of the Upper Pleistocene fauna 136,000 YBP. The last interglacial (MIS 5) on Bermuda also resulted in the extinction of all endemic birds, including a crane, a duck, and a flightless rail [22]. Therefore the presence of a reptile-rich deposit at Point Hodoul suggests that the atoll was subsequently recolonised by the above-named lizards, and some bird taxa, most notably a *Dryolimnas* rail. Giant tortoises appear to have recolonised Aldabra from Madagascar at least three times after these submergence events, presumably due to their ability to cover large expanses of open ocean by floating [1, 76]. If land refugia occurred elsewhere, e.g. Assumption Island, it could have required a shorter recolonisation distance than from Madagascar or the Comoros. It is not known for certain when the lizard fauna disappeared, because no Holocene fossil deposits exist, but the breaching of the Aldabra land rim and flooding of the lagoon around 5,000–7,000 YBP, which resulted in the loss of 60% of the land surface area, may have been the cause [1, 10].

Another interesting discovery is the presence of an unidentified *Circus* harrier in both of the Ile Picard deposits, representing at least three individuals, of which one is a skeletally associated individual. This suggests that a resident population was present on Aldabra in the Upper Pleistocene, especially during sea level low stands when the land surface area was greater. Furthermore, confirmation that a *Tyto* owl was also present in the older deposit of Bassin Cabri has added another predatory bird to the atoll’s faunal list. As the atoll was submerged between the Bassin Cabri and Point Hodoul deposits, owls must have colonised the atoll on at least two occasions during the Upper Pleistocene, as the rich owl-deposited lizard accumulations at Point Hodoul suggests that an owl was resident [1]; it is very likely that *Tyto alba* was involved on both occasions.

A recently reported fossil locality on Grand Terre contained giant tortoise and crocodile remains, all found loose on the surface in an area surrounding a pond [77]. The crocodile elements appear to belong to Nile Crocodile *Crocodylus niloticus*, and not to the endemic *Aldabrachampsus dilophus*, which suggests that Nile crocodile colonised Aldabra after the extinction of the latter. The authors state that the fossil material is Upper Pleistocene, but this is based on the Upper Pleistocene date for the Aldabra Limestone [8]. We suggest that as it was surface material, and not in matrix as was all other Upper Pleistocene material described here, it is very likely to be much younger, probably Holocene in age. Therefore, our conclusions are not affected by this discovery.

The fossiliferous deposits of Bassin Cabri and Bassin Lebine represent a small but significant window into the original faunal diversity of Aldabra during the Upper Pleistocene. The deposits are dominated by seabirds, with few terrestrial bird species, but an abundant terrestrial reptile fauna. Furthermore, the majority of species are derived from the Malagasy region, including the Comoros, and not from SE Asia as in the case of the Mascarenes [78]. Our present understanding of the geological and palaeontological history of Aldabra is not sufficient to determine the number of times the atoll was completely submerged during the Upper Pleistocene, but the Aldabra Limestone depositional event shows that at least one complete inundation event occurred. Therefore, it is likely, based on the fossil evidence presented here, that an almost complete faunal turnover occurred between the Bassin Cabri/Bassin Lebine and the Point Hodoul fossil deposits during the Upper Pleistocene, and again between the Upper Pleistocene and Holocene due to fluctuating sea levels.

## Supporting information

S1 File Data deposition. All fossil material in the text is deposited and accessioned in an appropriate public repository, the United States National Museum (USNM). All fossil material was loaned by the USNM for study and will be returned for permanent depository and public availability.
(DOCX)
