# Supplementary material for: A terrestrial vertebrate palaeontological review of Aldabra Atoll, Aldabra Group, Seychelles
Source: PLoS One. 2018 Mar 28;13(3):e0192675. doi: 10.1371/journal.pone.0192675 (PMC5873930; doi:10.1371/journal.pone.0192675)
Supplement: S1 File — All fossil material in the text is deposited and accessioned in an appropriate public repository, the United States National Museum (USNM). All fossil material was loaned by the USNM for study and will be returned for permanent depository and public availability. (DOCX) [file pone.0192675.s001.docx]

Supporting information

S1 File. Data deposition. All fossil material in the text is deposited and accessioned in an appropriate public repository, the United States National Museum (USNM). All fossil material was loaned by the USNM for study and will be returned for permanent depository and public availability.
